# Multiple Gene Clusters and Their Role in the Degradation of Chlorophenoxyacetic Acids in *Bradyrhizobium* sp. RD5-C2 Isolated from Non-Contaminated Soil

**DOI:** 10.1264/jsme2.ME21016

**Published:** 2021-09-11

**Authors:** Shohei Hayashi, Sho Tanaka, Soichiro Takao, Shinnosuke Kobayashi, Kousuke Suyama, Kazuhito Itoh

**Affiliations:** 1 Faculty of Life and Environmental Science, Shimane University, 1060 Nishikawatsu, Matsue, Shimane 690–8504, Japan

**Keywords:** 2,4-dichlorophenoxyacetic acid, 2,4,5-trichlorophenoxyacetic acid, multiplicities of the degradation gene, deletion mutant, gene expression

## Abstract

*Bradyrhizobium* sp. RD5-C2, isolated from soil that is not contaminated with 2,4-dichlorophenoxyacetic acid (2,4-D), degrades the herbicides 2,4-D and 2,4,5-trichlorophenoxyacetic acid (2,4,5-T). It possesses *tfdAα* and *cadA* (designated as *cadA1*), which encode 2,4-D dioxygenase and the oxygenase large subunit, respectively. In the present study, the genome of *Bradyrhizobium* sp. RD5-C2 was sequenced and a second *cadA* gene (designated as *cadA2*) was identified. The two *cadA* genes belonged to distinct clusters comprising the *cadR1A1B1K1C1* and *cadR2A2B2C2K2S* genes. The proteins encoded by the *cad1* cluster exhibited high amino acid sequence similarities to those of other 2,4-D degraders, while Cad2 proteins were more similar to those of non-2,4-D degraders. Both *cad* clusters were capable of degrading 2,4-D and 2,4,5-T when expressed in non-2,4-D-degrading *Bradyrhizobium elkanii* USDA94. To examine the contribution of each degradation gene cluster to the degradation activity of *Bradyrhizobium* sp. RD5-C2, *cadA1*, *cadA2*, and *tfdAα* deletion mutants were constructed. The *cadA1* deletion resulted in a more significant decrease in the ability to degrade chlorophenoxy compounds than the *cadA2* and *tfdAα* deletions, indicating that degradation activity was primarily governed by the *cad1* cluster. The results of a quantitative reverse transcription-PCR analysis suggested that exposure to 2,4-D and 2,4,5-T markedly up-regulated *cadA1* expression. Collectively, these results indicate that the *cad1* cluster plays an important role in the degradation of *Bradyrhizobium* sp. RD5-C2 due to its high expression.

Since the 1940s, chlorophenoxy herbicides, such as 2,4-dichlorophenoxyacetic acid (2,4-D) and 2,4,5-trichlorophenoxyacetic acid (2,4,5-T), have been widely used to control the growth of broadleaf weeds. These two herbicides were the main components of Agent Orange, which was sprayed during the Vietnam War. 2,4-D is still used worldwide, and is a model compound for studying the microbial acquisition of genes capable of degrading anthropogenic chemicals and the distribution of genes within a microbial genome. Diverse 2,4-D-degrading bacteria, belonging to *Actinobacteria*, *Bacteroidetes*, and *Alpha-*, *Beta-*, and *Gammaproteobacteria* phyla, have been isolated from various environments. Although 2,4,5-T has been prohibited worldwide due to its toxicity to humans, residues of 2,4,5-T have been reported in Canada, USA, and Vietnam (Rice *et al.*, 2005; Donald *et al.*, 2007; Huong *et al.*, 2007b). Since 2,4,5-T is more persistent than 2,4-D, fewer studies have been conducted on 2,4,5-T-degrading bacteria than on 2,4-D degraders (Kilbane *et al.*, 1982; Golovleva *et al.*, 1990; Rice *et al.*, 2005; Huong *et al.*, 2007b).

2,4-D-degrading bacteria typically possess the *tfdA* (*tfdAα*), *tftAB*, and/or *cadAB*(*C*) genes, which catalyze the first step of the degradation pathway (Nojiri *et al.*, 2014; Serbent *et al.*, 2019). The proteins encoded by *tfdA*, *tftAB*, and *cadABC* catalyze the transformation of 2,4-D into 2,4-dichlorophenol (2,4-DCP). *tfdA* encodes an α-ketoglutarate-dependent dioxygenase (TfdA), *tftA* and *cadA* encode oxygenase large subunits (TftA and CadA, respectively), *tftB* and *cadB* encode small subunits (TftB and CadB, respectively), and *cadC* encodes a ferredoxin component (CadC). Dichlorophenol hydroxylase (TftB) then converts 2,4-DCP into 3,5-dichlorocatechol, which is further degraded via a modified *ortho*-cleavage pathway (Liu and Chapman, 1984; Perkins *et al.*, 1990; Laemmli *et al.*, 2000). Briefly, the cleavage of 3,5-dichlorocatechol by chlorocatechol 1,2-dioxygenase (TfdC) forms 2,4-dichloro-*cis*-*cis*-muconate. This is converted to 2-chlorodienelactone by chloromuconate cycloisomerase (TfdD), which is then transformed to 2-chloromaleylacetate by chlorodienelactone hydrolase (TfdE). 2-Chloromaleylacetate is converted by chloromalelylacetate reductase (TfdF), through the formation of maleylacetate, into beta-ketoadipate, which then enters the tricarboxylic acid cycle (Laemmli *et al.*, 2000; Kumar *et al.*, 2016b; Serbent *et al.*, 2019).

Although the induction of *cadABC* remains unclear, *cadR* in the 2,4-D degrader *Bradyrhizobium* sp. HW13 was shown to be essential for the heterologous expression of *cadA* (Kitagawa *et al.*, 2002). In contrast, in the non-2,4-D degrader *Bradyrhizobium elkanii* USDA94, *cadR* did not lead to the downstream induction of *cadABC* even when 2,4-D was present in the culture medium (Hayashi *et al.*, 2016).

Various 2,4,5-T-degrading bacteria, including *Burkholderia* spp. (Kellogg *et al.*, 1981; Danganan *et al.*, 1994; Xun and Wagnon, 1995; Huong *et al.*, 2007b), *Nocardioides simplex* 3E (Golovleva *et al.*, 1990), *Sphingomonas* spp. (Huong *et al.*, 2007b), and *Bradyrhizobium* spp. (Rice *et al.*, 2005; Huong *et al.*, 2007b), have been reported. Previous studies on degradation genes demonstrated that TftAB and CadABC have the ability to convert 2,4,5-T to 2,4,5-trichlorophenol (2,4,5-TCP) (Danganan *et al.*, 1994; Kitagawa *et al.*, 2002; Hayashi *et al.*, 2016).

The multiplicities of *cad* genes responsible for 2,4-D and 2,4,5-T degradation have not yet been reported. However, previous studies showed the multiplicities of genes that encode degrading enzymes for xenobiotic compounds. For example, *Cupriavidus pinatubonensis* JMP134 contains duplicate *tfdBCDEF* gene clusters for chlorophenol degradation (Leveau *et al.*, 1999), *Sphingomonas* sp. KA1 encodes two distinct *car* clusters for carbazole degradation (Urata *et al.*, 2006), *Rhodococcus jostii* RHA1 possesses three chlorobiphenyl 2,3-dioxygenase genes (Iwasaki *et al.*, 2006), and *Mycobacterium* spp. harbor several pyrene-degrading gene clusters (Sho *et al.*, 2004; Kim *et al.*, 2006; Zhang and Anderson, 2012). Two methods generally lead to multiplicities in catabolic genes-gene duplication in the genome and horizontal gene transfer from outside sources. The multiplicities of catabolic genes may promote adaption to the use of novel sources in the environment (Gevers *et al.*, 2004). The study of genes involved in the degradation of xenobiotic compounds has provided insights into the acquisition of this ability in several environments. The information obtained has contributed to a more detailed understanding of how and why bacteria adapt and evolve to acquire the ability to degrade xenobiotic compounds. The multiplicities of related genes are considered to be one step in the process of the acquisition of this ability.

We previously isolated *Bradyrhizobium* sp. RD5-C2, a 2,4-D-degrading strain, from arable soil in Japan with no history of exposure to 2,4-D (Itoh *et al.*, 2000). This strain possesses a *cadA* gene (designated as *cadA1* in the present study), which is highly similar to the *cadA* gene of another 2,4-D degrader, *Bradyrhizobium* sp. HW13 (Fig. 1) (Itoh *et al.*, 2004). It also possesses the *tfdAα* gene, which exhibits weak 2,4-D dioxygenase activity when expressed in *Escherichia coli* (Itoh *et al.*, 2002). The purpose of the present study was to obtain genetic information on the degradation genes present in *Bradyrhizobium* sp. RD5-C2 and elucidate the role of the aforementioned three degradation genes in the degradation of 2,4-D and 2,4,5-T.

## Materials and Methods

### Identification and phylogenetic analysis of *cad* clusters and *tfdAα* in *Bradyrhizobium* sp. RD5-C2

Draft genome sequencing of *Bradyrhizobium* sp. RD5-C2 was performed using Illumina HiSeq2000 equipment (Illumina). The *de novo* assembly of the resulting sequence data was performed using Velvet software version 1.2.08. Two distinct *cad* clusters, designated as *cad1* and *cad2*, and *tfdAα* were identified in the genome using the sequences of *cadA* and *tfdAα* from *Bradyrhizobium* sp. RD5-C2 (accession no. AB119238 and AB074490) and *B. elkanii* USDA94 (AB119244), respectively, with the MUMmer 3.23 software program (Kurtz *et al.*, 2004). Putative ORFs were identified using the Joint Genome Institute portal (http://jgi.doe.gov/), and phylogenetic trees were constructed using the neighbor-joining method with the MEGA7 software program (Kumar *et al.*, 2016a). The nucleotide sequences identified in the present study have been annotated using DFAST and deposited in the DNA Data Bank of Japan (DDBJ, http://www.ddbj.nig.ac.jp/index-j.html); accession numbers for the draft genome sequences are BOVL01000001 to BOVL01000073. The locus tags of *cadR1A1B1K1C1*, *cadR2A2B2C2K2S*, and *tfdAα* are BraRD5C2_67200 to BraRD5C2_67240, BraRD5C2_72670 to BraRD5C2_72620, and BraRD5C2_05110, respectively. All genes used in the present study are listed in Table S1.

### Bacterial strains, plasmids, and growth conditions

All *Bradyrhizobium* strains, plasmids, and primers used in the present study are listed in Table 1, S2, and S3. *Bradyrhizobium* strains were cultivated in HM medium at 25°C (Minamisawa *et al.*, 1998), and *E. coli* strains were manipulated as previously described (Sambrook and Russell, 2001). *E. coli* S17-1λpir (Mazodier *et al.*, 1989) was used as the transconjugation donor, and *E. coli* transformants were grown in Luria-Bertani (LB) medium (Sambrook and Russell, 2001) with appropriate antibiotics at 37°C.

### Cloning of *cadA1B1K1C1* and *cadA2B2C2K2* into *B. elkanii* USDA94

To examine the degradation activities of *cadA1B1K1C1* and *cadA2B2C2K2* in a related strain of *Bradyrhizobium* sp. RD5-C2, the clusters were expressed in the non-2,4-D degrader, *B. elkanii* USDA94. The *cadA1B1K1C1* fragment was amplified with PCR using KOD plus DNA polymerase (Toyobo) with the primer set of C2cad1A-F-Nde/C2cad1C-R-Bam. The PCR amplification mixture was prepared according to the manufacturer’s instructions. The amplification reaction was as follows: 94°C for 2‍ ‍min, followed by 30 cycles at 95°C for 15‍ ‍s, 60°C for 15‍ ‍s, and 68°C for 6.5‍ ‍min. The amplified DNA fragment was then digested with *Nde*I and *Bam*HI (FastDigest, Thermo Fisher Scientific) and inserted into the multiple cloning site of pBBR1MCS2_START (Obranić *et al.*, 2013) to yield pBBR2-C2cad1ABKC. *cadA2B2C2K2* and pBBR1MCS2_START fragments were amplified with PCR using KOD plus neo DNA polymerase (Toyobo) with the primer sets BBR2+C2cad2-F/BBR2+C2cad2-R and C2cad2+BBR2-F/C2cad2+BBR2-R, respectively. The amplification reaction was as follows: 94°C for 2‍ ‍min followed by 30 cycles at 98°C for 10‍ ‍s, 64°C (*cadA2B2C2K2*) or 62°C (pBBR1MCS2_START) for 30‍ ‍s, and 68°C for 2‍ ‍min (*cadA2B2C2K2*) or 2‍ ‍min and 45‍ ‍s (pBBR1MCS2_START). The amplified fragments were assembled to yield pBBR2-C2cad2ABKC using the NEBuilder HiFi DNA Assembly Master Mix (New England Biolabs) according to the manufacturer’s instructions. The constructed plasmids were cloned into *E. coli*, and the transformants were cultivated on LB agar medium supplemented with kanamycin (30‍ ‍mg L^–1^). The fidelity of inserts was confirmed with nucleotide sequencing. The extracted plasmid was transformed into *E. coli* S17-1λpir and subsequently introduced into *B. elkanii* USDA94 using conjugative transformation, as previously described (Hayashi *et al.*, 2016).

### Chlorophenoxyacetic acid-degrading activities of *B. elkanii* transformants

*Bradyrhizobium* transformants were cultivated in HM medium containing 100‍ ‍μM 2,4-D or 100‍ ‍μM 2,4,5-T and kanamycin (150‍ ‍mg L^–1^) at 25°C with shaking. At appropriate intervals, the concentrations of compounds and degradation products (2,4-DCP and 2,4,5-TCP) in the supernatant were measured using a Prominence ultra-fast liquid chromatography system (Shimadzu) equipped with an SPD-M20A photodiode array (Shimadzu) and Shim-pack XR-ODS column (2.2‍ ‍μm, 100‍ ‍mm length×3.0‍ ‍mm i.d., Shimadzu), as previously described (Hayashi *et al.*, 2016).

### Construction of *Bradyrhizobium* sp. RD5-C2 *cadA1*, *cadA2*, *tfdAα*, and *cadR1* deletion mutants

To produce in-frame deletion mutants of *cadA1*, *cadA2*, and *tfdAα*, insertional inactivation via double crossover was performed as previously described (Hayashi *et al.*, 2016). The upstream and downstream regions of each gene were PCR-amplified using the following primer sets: Dcad1Aup5-Eco/Dcad1Aup3-Xba and Dcad1Adw5-Xba/Dcad1Adw3-Hind for *cadA1*; DcadAup5-Kpn/DcadAup3-Xba and DcadAdw5-Xba/DcadAdw3-Hind for *cadA2*; DtfdAαup5-Kpn/DtfdAαup3-Xba and DtfdAαdw5-Xba/DtfdAαdw3-Hind for *tfdAα*. The digested fragments were ligated into the multiple cloning sites of pK18mob (Schäfer *et al.*, 1994) to generate pK18mob-C2cadA1updw, pK18mob-C2cadA2updw, and pK18mob-C2tfdAαupdw with in-frame deletions in *cadA1*, *cadA2*, and *tfdAα*, respectively. The resulting plasmids were introduced into *Bradyrhizobium* sp. RD5-C2 via *E. coli* S17-1λpir. Double-crossover mutants were screened from single crossover mutants based on kanamycin sensitivity. Successful in-frame deletions of 1,011 bp in *cadA1*, 1,038 bp in *cadA2*, and 634 bp in *tfdAα* were confirmed by the sequencing of new junction regions. To construct *cadA1* and *cadA2* double-deletion mutants, the 4-kb fragment in pK18mob-C2cadA1updw was amplified using the primers DcadAup5-Bam/DcadAdw3-Hind and then cloned into the multiple cloning sites of pK18mobsacB (Schäfer *et al.*, 1994) to yield pK18mobsacB-C2cadA2updw, which was cloned into *Bradyrhizobium* sp. RD5-C2ΔcadA1 to delete the *cadA2* gene. To generate *cadA1*, *cadA2*, and *tfdAα* triple-deletion mutants, the *tfdAα* gene of *Bradyrhizobium* sp. RD5-C2ΔcadA1ΔcadA2 was deleted using pK18mobsacB-C2tfdAαupdw, which contains the PCR-amplified fragment of pK18mob-94tfdAαupdw, using the primers DtfdAaup5-Bam/DtfdAαdw3-Hind. Double-crossover mutants were screened by culturing on HM medium containing 5% sucrose, which kills cells that containing the *sacB* (levansucrase) gene derived from pK18mobsacB (Schäfer *et al.*, 1994), and using kanamycin sensitivity. To construct a *cadR1* deletion mutant, the upstream and downstream regions of *cadR1* were amplified using the primer sets DC2cad1up5-Hin/DC2cad1up3-Xba and DC2cad1Rdw5-Xba/DC2cad1Rdw3-Bam, respectively. Digested fragments were ligated into the multiple cloning sites of pK18mobsacB (Schäfer *et al.*, 1994) to yield pK18mobsacB-C2cadR1updw. The deletion of *cadR1* was conducted as described above for the *cadA1*, *cadA2* and *tfdAα* deletions.

To construct the complementary strain of the *cadA1* deletion mutant, *cadA1* was expressed under the control of the *cadA1* promoter because our preliminary experiments indicated that the *lac* promoter did not induce the expression of downstream genes in *Bradyrhizobium* sp. RD5-C2 (data not shown). The fragment containing the *cadA1* and *cadA1* promoter regions was amplified with PCR using KOD plus DNA polymerase (Toyobo) with the primer set C2cad1P-F-Mph/C2cadA1-Bam-R. The resultant fragment was digested with *Mph*11031 and *BamH*I (FastDigest, Thermo Fisher Scientific), and ligated into pBBR1MCS2_START to yield pBBR2-C2cadA1pro-cadA1. The constructed plasmid was cloned into *E. coli*, amplified, and extracted. The plasmid was then transformed into *E. coli* S17-1λpir and introduced into the *cadA1* deletion mutant to generate the complementary strain, as described above for the introduction of *cad* clusters into *B. elkanii* USDA94. The complementary strain was cultivated in HM medium supplemented with kanamycin (150‍ ‍mg L^–1^).

### Analysis of expression levels of degradation genes using quantitative reverse transcription-PCR (qRT-PCR)

After *Bradyrhizobium* sp. RD5-C2 was precultivated in HM medium to reach the stationary phase, it was exposed to 100‍ ‍μM 2,4-D (1 day) and 100‍ ‍μM 2,4,5-T (1 and 3 days) with shaking. Total RNA was extracted using ISOGEN-LS (Nippon Gene) according to the manufacturer’s instructions. RNA samples were then treated with DNase I (Takara Bio), and 1‍ ‍μg of each treated sample was used for cDNA synthesis using the PrimeScript RT reagent Kit with gDNA Eraser (Takara Bio). Real-time PCR was performed using FastStart Essential DNA Green Master (Roche Diagnostics) and LightCycler Nano Instrument (Roche Diagnostics) according to the manufacturer’s instructions. The *sig* gene for sigma factor was used as an internal control. Primer sets were C2cad1A227-f/C2cad1A436-r (*cadA1*, 210‍ ‍bp), C2cad2A548-f/C2cad2A779-r (*cadA2*, 232‍ ‍bp), C2tfdAa308-f/C2tfdAa488-r (*tfdAa*, 181‍ ‍bp), and C2sig1288-f/C2sig1454-r (*sig*, 167‍ ‍bp). The PCR reaction was as follows: 95°C for 10‍ ‍min followed by 45 cycles at 95°C for 10‍ ‍s, 57°C for 10‍ ‍s, and 72°C for 15 s. The expression level of each gene was normalized to that of the *sig* (sigma factor) gene using the 2^–ΔCT^ (ΔCT=Ct target gene–CT sig) calculation for statistical analyses (Dunnett’s test [*P*<0.05]). Three biological experiments were conducted for each treatment, and three real-time PCR reactions were performed for each experiment.

## Results

### Two *cad* clusters and *tfdAα* identified in *Bradyrhizobium* sp. RD5-C2

The genome of *Bradyrhizobium* sp. RD5-C2 was sequenced using the Illumina HiSeq2000 platform. The preprocessing and assembly of 31,473,568 paired reads yielded 73 contigs, with a combined size of 8,259,668 bp and GC content of 64.2%. The completeness value of the draft genome was found to be 99.3% using CheckM (Parks *et al.*, 2015). The 16S ribosomal RNA, tRNA-Ile, tRNA-Ala, and 23S ribosomal RNA sequences of *Bradyrhizobium* sp. RD5-C2 showed 99% similarities to those of *Bradyrhizobium elkanii* USDA4341 (JQ911628).

**Fig. 1.**
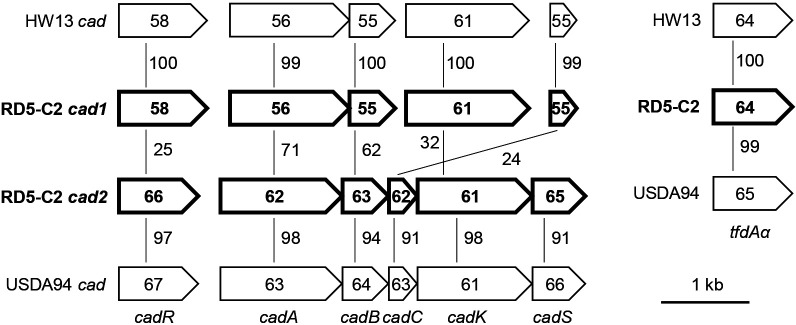
Comparison of *cad* clusters and *tfdAα* genes from *Bradyrhizobium* sp. HW13, *Bradyrhizobium* sp. RD5-C2, and *Bradyrhizobium elkanii* USDA94. Each gene is represented by a large horizontal arrow containing its GC content (mol %). Similarity values (%) of the deduced amino acid sequences of corresponding proteins are represented with thin lines. Genes from *Bradyrhizobium* sp. RD5-C2 are indicated in bold.

The nucleotide sequence around *cadA1* was elucidated, and a *cad1* cluster was identified. Additionally, a *cad2* cluster was identified within a different contig containing the *cad1* cluster. Based on the deduced amino acid sequences of putative ORFs, both *cad* clusters contained genes encoding a transcriptional regulator (*cadR*), the oxygenase large and small subunits (*cadA* and *cadB*, respectively), a ferredoxin component (*cadC*), and a transporter (*cadK*) (Fig. 1). An additional gene (*cadS*) encoding a transcriptional regulator was observed within the downstream region of *cadK2*. Amino acid sequence similarities were moderate (24–71%) between the corresponding Cad1 and Cad2 proteins. CadR1A1B1K1C1 showed high similarities (99–100%) with the corresponding proteins of the 2,4-D degrader *Bradyrhizobium* sp. HW13, while the sequences of CadR2A2B2C2K2S were similar (91–98%) to the non-2,4-D degrader *B. elkanii* USDA94. Similar results were observed for the GC contents of all genes, except *cadK*. The GC content of *cadR1A1B1C1* (55–58%) was lower than the average GC content (64.2%) of the genome. In the hierarchical cluster analysis of the codon usage of *cad* and 34 housekeeping genes, *cadR1A1B1K1* separated from other genes (Table S4 and Fig. S1). In contrast to the representative *tfdA* clustered with other *tfd* genes (Don and Pemberton, 1981; Leveau *et al.*, 1999), a corresponding gene was not detected around *tfdAα*, similar to *B. elkanii* USDA94 (Hayashi *et al.*, 2016). The *tfdAα* GC contents, *tfdAα* codon usage, and TfdAα sequences of the three *Bradyrhizobium* strains were equivalent (Fig. 1).

**Fig. 2.**
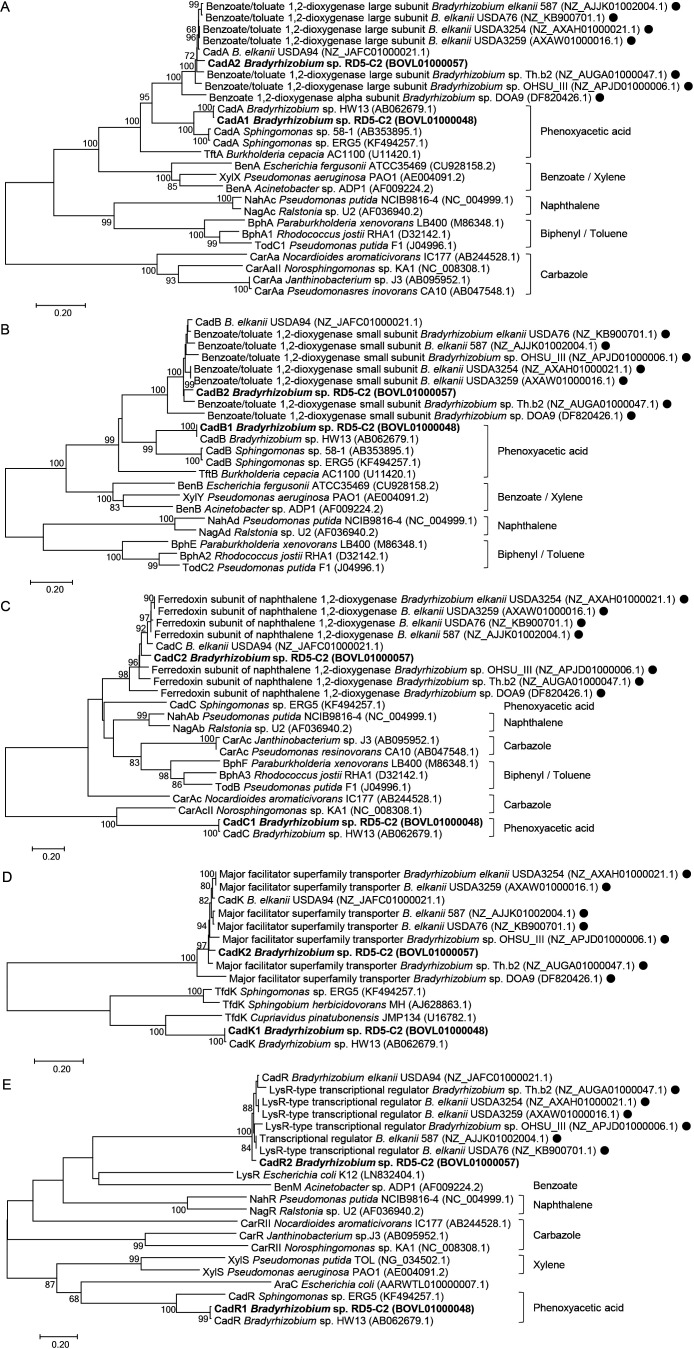
Phylogenetic tree analysis of CadA(A), CadB(B), CadC(C), CadK(D), and CadR(E). Phylogenetic trees were constructed for the amino acid sequences of CadA and CadB with related oxygenase large and small subunits, respectively, CadC with related ferredoxin components of oxygenases, CadK with related transporters, and CadR with related regulators using the Neighbor Joining method and 1,000 bootstrap replicates, constructed using the MEGA7 software program. Bootstrap values above 60% are shown at the nodes. Sequences from *Bradyrhizobium* sp. RD5-C2 are indicated in bold. Closed circles indicate homologs of Cad enzymes in the genomes of *Bradyrhizobium* strains. The representative substrates for each enzyme are indicated to the right of the figures. Scale bars indicate substitutions per site. The numbers on the right are accession numbers.

Phylogenetic trees were generated for Cad proteins and related enzymes, including Cad homologs in the genomes of *Bradyrhizobium* strains (Fig. 2). All corresponding Cad1 and Cad2 proteins were separated; the former were grouped into clades with those of *Bradyrhizobium* sp. HW13, while the latter formed distinct clades with other *Bradyrhizobium* strains. CadA1 and CadB1 in 2,4-D-degrading *Bradyrhizobium* were grouped with the corresponding Cad proteins in 2,4-D-degrading *Sphingomonas* (Müller *et al.*, 2004; Shimojo *et al.*, 2009; Nielsen *et al.*, 2013). CadA2 and CadB2 belonged to the clades containing related dioxygenases, which were annotated as benzoate/toluene 1,2-dioxygenase large and small subunits, respectively.

CadA1 and CadB1 exhibited 57 and 46% amino acid sequence similarities with the TftA and TftB proteins of *Burkholderia cepacia* AC1100 (Kellogg *et al.*, 1981), respectively, which degrade 2,4,5-T into 2,4,5-TCP (Danganan *et al.*, 1994; Xun and Wagnon, 1995). CadA2 and CadB2 were 54 and 48% similar to TftA and TftB, respectively. CadA and CadB in *Bradyrhizobium* spp. were separated from oxygenases involved in the degradation of aromatic compounds, including BenA and BenB (benzoate) of *Acinetobacter* sp. ADP1 (Neidle *et al.*, 1991), NahAc and NahAd (naphthalene) of *Pseudomonas putida* NCIB9816-4 (Dennis and Zylstra, 2004), TodC1 and TodC2 (toluene) of *P. putida* F1 (Zylstra and Gibson, 1989), and CarAa (carbazole) of *Nocardioides aromaticivorans* (Inoue *et al.*, 2006) (Fig. 2A and B).

In the phylogenetic tree of CadC, CadC1 and CadC of *Bradyrhizobium* sp. HW13 formed an independent clade with CarAcII, a ferredoxin component of carbazole 1,9a-dioxygenase from *Norosphingomonas* sp. KA1 (Urata *et al.*, 2006) (Fig. 2C). They were separated from the CadC of *Sphingomonas* sp. ERG5 (Nielsen *et al.*, 2013) and the related ferredoxins involved in the degradation of aromatic compounds. CadK1 formed a distinct clade with TfdK from *Cupriavidus necator* JMP134, *Sphingomonas* sp. ERG5 (Nielsen *et al.*, 2013), and *Sphingomonas herbicidovorans* MH (Müller *et al.*, 2004) (Fig. 2D). CadR1 was grouped in an independent clade with XylS from *Pseudomonas* spp. (Gomada *et al.*, 1992; Stover *et al.*, 2000) and belonged to an AraC-type transcriptional regulator (Fig. 2E). On the other hand, CadR2 formed a clade containing BenM from *Acinetobacter* sp. ADP1 (Collier *et al.*, 1998), NahR from *P. putida* NCIB9816-4 (Dennis and Zylstra, 2004), and NagR from *Ralstonia* sp. U2 (Zhou *et al.*, 2001), which are LysR-type transcriptional regulators.

### Genes for the degradation of 2,4-DCP and 2,4,5-TCP

*tfdBaFRDEC* genes were identified upstream of the *cad1* cluster and three ORFs were detected between *tfdBa* and other *tfd* genes (Fig. S2). An analysis of the deduced amino acid sequences of *tfdBaFRDEC* indicated that they were 2,4-DCP 6-monooxygenase, maleylacetate reductase, LysR family transcriptional regulator, TfdD, TftB, and TfdC. There were five catabolic genes for the conversion of chlorophenol before it entered the tricarboxylic acid cycle. Their GC contents (54–56%) were similar to those of *cad1* genes and different from the average GC content of the entire genome.

### Degradation of chlorophenoxyacetic acids by *cad* cluster transformants

**Fig. 3.**
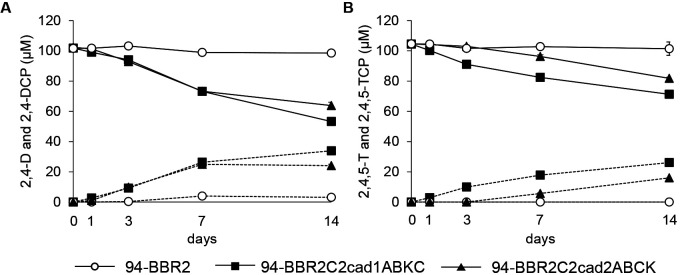
Degradation of 2,4-dichlorophenoxyacetic acid (2,4-D) (A) and 2,4,5-trichlorophenoxyacetic acid (2,4,5-T) (B) by *Bradyrhizobium elkanii* USDA94 harboring *cadA1B1K1C1* and *cadA2B2C2K2* from *Bradyrhizobium* sp. RD5-C2. Solid and dashed lines indicate substrates and their corresponding degradation products (2,4-dichlorophenol [2,4-DCP] and 2,4,5-trichlorophenol [2,4,5-TCP]), respectively. Error bars indicate standard deviations based on triplicate cultures. If not visible, error bars are smaller than symbols.

*B. elkanii* USDA94-BBR2C2cad1ABKC degraded 30 and 20% of 2,4-D and 2,4,5-T, respectively, in 7 days, and the corresponding degradation products were detected (Fig. 3). *B. elkanii* USDA94-BBR2C2cad2ABCK degraded 30 and 8% of 2,4-D and 2,4,5-T, respectively. The 2,4-D degradation rate of *B. elkanii* USDA94-BBR2C2cad1ABKC was similar to that of *B. elkanii* USDA94-BBR2C2cad2ABCK. The 2,4,5-T degradation rate of the former was faster than that of the latter. The control strain, *B. elkanii* USDA94-BBR2, showed negligible or no degrading activity for 2,4-D or 2,4,5-T, as previously reported (Hayashi *et al.*, 2016).

### Degradation of chlorophenoxyacetic acids by *cadA1*, *cadA2*, and *tfdAα* deletion mutants

**Fig. 4.**
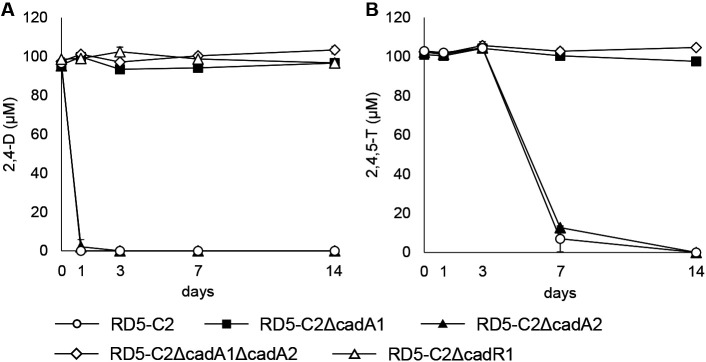
Degradation of 2,4-dichlorophenoxyacetic acid (2,4-D) (A) and 2,4,5-trichlorophenoxyacetic acid (2,4,5-T) (B) by *cadA1* and/or *cadA2* deletion mutants of *Bradyrhizobium* sp. RD5-C2. Error bars indicate standard deviations based on triplicate cultures. If not visible, error bars are smaller than symbols.

The wild-type strain *Bradyrhizobium* sp. RD5-C2 degraded 2,4-D and 2,4,5-T, and no degradation products were detected (Fig. 4). While 2,4-D disappeared within 1 day of the incubation, 2,4,5-T concentrations began to decrease after 3 days, and a small amount of the compound was detected after 7 days. *Bradyrhizobium* sp. RD5-C2ΔcadA2 (Fig. 4) and *Bradyrhizobium* sp. RD5-C2ΔtfdAα (Fig. S3) degraded 2,4-D and 2,4,5-T similar to the wild-type strain. On the other hand, degradation by *Bradyrhizobium* sp. RD5-C2ΔcadA1 was negligible. The double-deletion mutant, *Bradyrhizobium* sp. RD5-C2ΔcadA1ΔcadA2 (Fig. 4), and the triple-deletion mutant, *Bradyrhizobium* sp. RD5-C2ΔcadA1ΔcadA2ΔtfdAα (Fig. S3), did not degrade 2,4-D or 2,4,5-T. *Bradyrhizobium* sp. RD5-C2ΔcadR1 only slightly degraded 2,4-D, similar to *Bradyrhizobium* sp. RD5-C2ΔcadA1. Negligible and no degradation were confirmed in comparisons with non-inoculated samples (data not shown). Although the degradation rate of the complementary strain did not equal that of the wild-type strain, it was faster than *Bradyrhizobium* sp. RD5-C2ΔcadA1/pBBR2, which had the empty vector introduced in *Bradyrhizobium* sp. RD5-C2ΔcadA1, indicating that 2,4-D-degrading activity was complemented by the introduction of *cadA1* under the control of the *cadA1* promoter (Fig. S4).

### Expression of degradation genes in *Bradyrhizobium* sp. RD5-C2 following exposure to chlorophenoxyacetic acids

**Fig. 5.**
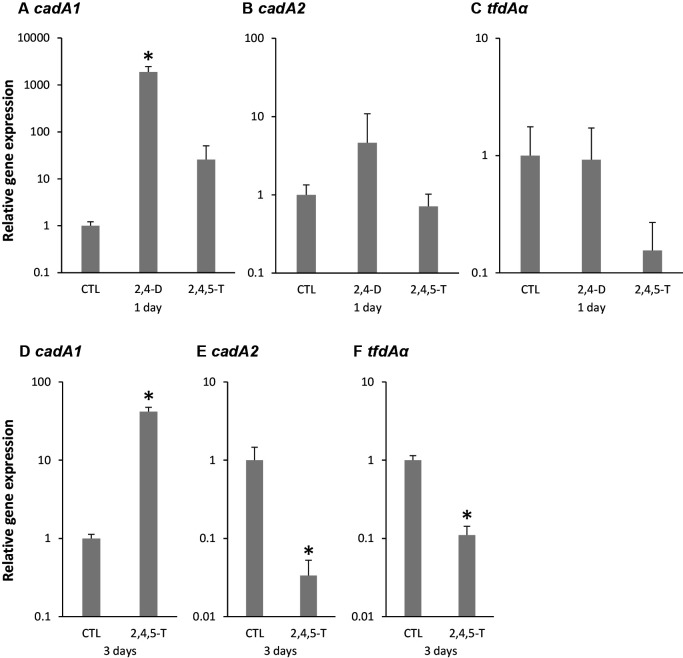
Effects of a 1- and 3-day exposure to 2,4-dichlorophenoxyacetic acid (2,4-D) and 2,4,5-trichlorophenoxyacetic acid (2,4,5-T) on the expression of *cadA1* (A and D), *cadA2* (B and E), and *tfdAα* (C and F) in *Bradyrhizobium* sp. RD5-C2. The expression level of each gene was normalized to that of the *sig* (sigma factor) gene. CTL indicates the control condition. Error bars indicate standard deviations based on triplicate cultures. The means of the control condition in triplicate cultures are shown as 1. Statistical analyses were performed for comparisons with the control (Dunnett’s test, *n*=3, *P<0.05).

A significantly higher *cadA1* expression level was detected following exposure to 2,4-D than under the control condition (Fig. 5). The average relative expression of *cadA1* was more than 1,000-fold higher than that under the control condition. *cadA1* expression after 1 day of exposure to 2,4,5-T did not significantly differ from that under the control condition; however, its average relative expression was more than 10-fold higher. The expression levels of *cadA2* and *tfdAα* after 1 day of exposure to 2,4-D and 2,4,5-T did not significantly differ from those under the control condition. A significantly higher expression level of *cadA1* and lower expression levels of *cadA2* and *tfdAα* were detected 3 days after exposure to 2,4,5-T than under the control condition (Fig. 5). *cadA1* expression levels did not markedly vary in the three biological replicants. No specific fragment was obtained without RT-PCR, indicating that the samples were not contaminated with DNA (data not shown).

## Discussion

### Roles of *cad1*, *cad2*, and *tfdAα* in the degradation of chlorophenoxyacetic acids

In the present study, two *cad* clusters with distinctly different phylogenies were identified in the genome of *Bradyrhizobium* sp. RD5-C2. Although both *cad* clusters possessed the ability to degrade 2,4-D and 2,4,5-T, the *cad1* cluster was mainly responsible for their degradation. qRT-PCR analyses revealed that the contribution of the *cad1* cluster to the degradation of 2,4-D was attributed to the high induction of the *cad1* cluster following the exposure to 2,4-D. The expression of the *cad1* cluster exposed to 2,4,5-T was significantly higher than that under the control condition after 3 days of exposure, suggesting that the induction of the *cad1* cluster by 2,4,5-T required a longer time. This coincides with the 3-day lag before the initiation of 2,4,5-T degradation (Fig. 4B). CadR1 is assumed to be important for the expression of *cad1*-degrading genes and their functions in the degradation of chlorophenoxyacetic acids.

Although the degradation rate of the complementary strain did not equal that of the wild-type strain, it was faster than that of RD5-C2ΔcadA1/BBR2 (Fig. S4). A previous study reported that the *benA* complementary strain of *Rhodococcus* sp. RHA1, which uses benzoate as a sole carbon source, grew on benzoate; however, its growth rate was lower than that of the wild type (Kitagawa *et al.*, 2001). The gene *benA* encodes the benzoate dioxygenase large subunit and forms an operon with *benB*, which encodes the benzoate dioxygenase small subunit. The genes *cadA1* and *cadB1* are most likely transcribed as a single operon when the start codon of *cadB1* overlaps with the upstream region of the termination codon of *cadA1*, and no promoter sequence was detected upstream of *cadB1*. To avoid a polar effect, the *cadA1* deletion mutant was constructed without changing the triplet sequences downstream. Based on the recovery of 2,4-D degradation activity following the introduction of *cadA1* and construction of deletion mutants, we concluded that the *cadA1* deletion significantly decreased the 2,4-D degradation rate.

The effects of the *cadA2* deletion were only observed in the *cadA1* and *cadA2* double-deletion mutant (Fig. 4), suggesting that although the *cad2* cluster exhibits similar degradation activity to the *cad1* cluster (Fig. 3), the contribution of the *cad2* cluster was very small. The expression of *cadA2* after a 1-day exposure to 2,4-D and 2,4,5-T did not significantly differ from that under the control condition, while a 3-day exposure to 2,4,5-T significantly reduced the expression of *cadA2*. This result indicates that the *cad2* cluster was not induced by 2,4-D or 2,4,5-T and also that a longer exposure to 2,4,5-T inhibited the expression of the *cad2* cluster, which may explain why the *cad2* cluster was not primarily responsible for degradation. *B. elkanii* USDA94-BBR2C2cad2ABCK degraded 2,4-D similar to *B. elkanii* USDA94-BBR2C2cad1ABKC (Fig. 3A). Therefore, if the *cad2* cluster is expressed at a similar level to the *cad1* cluster in *Bradyrhizobium* sp. RD5-C2, it may play an equivalent role in degradation to the *cad1* cluster.

The effects of the *tfdAα* deletion were not detected in the degradation of 2,4-D or 2,4,5-T (Fig. S3), and the *tfdAα* deletion mutant degraded 4-chlorophenoxyacetic acid and phenoxyacetic acid similar to the wild-type strain (data now shown). The TfdAα protein expressed in *E. coli* exhibited degradation activities for 2,4-D, 4-chlorophenoxyacetate, and phenoxyacetate *in vitro* (Itoh *et al.*, 2002). The forced expression of *tfdAα* in *B. elkanii* USDA94, which is very similar to *tfdAα* in *Bradyrhizobium* sp. RD5-C2, did not lead to an increase in 2,4-D degradation (Hayashi *et al.*, 2016). The expression level of *tfdAα* after a 3-day exposure to 2,4,5-T was significantly lower than that under the control condition, indicating that 2,4,5-T inhibited the expression of *tfdAα*. These results suggest that *tfdAα* does not play a significant role in the xenobiotic degradation activity of *Bradyrhizobium* sp. RD5-C2, whereas the TfdAα protein degrades (chloro)phenoxyacetic acids *in vitro*.

The 2,4-DCP conversion by TfdBa (Huong *et al.*, 2007a) and the results of the analysis of the deduced amino acid sequence of *tfdBaFDEC* in the genome of *Bradyrhizobium* sp. RD5-C2 indicate that the *tfd* genes play a role in the degradation of 2,4-DCP by the same pathway as in *C. pinatubonensis* JMP134. This is supported by the finding showing that *Bradyrhizobium* sp. RD5-C2 uses 2,4-D as a sole carbon and energy source (Itoh *et al.*, 2000). The protein encoded by *tfdR* is a transcriptional regulator that controls the expression of other *tfd* genes. *tfdBaCDEF* is presumed to play a role in the degradation of 2,4,5-TCP because 2,4,5-TCP was not detected in the culture during 2,4,5-T degradation.

### Multiplicities of chlorophenoxyacetic acid degradation genes

A previous study reported that *R. jostii* RHA1 possessed three chlorobiphenyl 2,3-dioxygenase genes, *bphA1*, *etbA1*, and *ebdA1* (Iwasaki *et al.*, 2006). The 4-chlorobiphenyl-degrading activity of the single insertion mutants of dioxygenase genes indicated that all were involved in degradation. Sho *et al*. (2004) reported that *Mycobacterium* sp. S65 possessed two pyrene- and phenanthrene-degrading gene clusters (*nid* and *pdo* clusters), and both clusters were transcribed with pyrene and phenanthrene. In contrast to these strains, only one of the three genes, the *cad1* cluster, was expressed, and it degraded chlorophenoxyacetic acids in *Bradyrhizobium* sp. RD5-C2. The multiplicities of the degradation gene homologues of xenobiotic compounds in a strain have been reported for chlorophenol in *C. pinatubonensis* JMP134 (Leveau *et al.*, 1999), carbazole in *Norosphingomonas* sp. KA1 (Urata *et al.*, 2006), and dihydroxybiphenyl in *Rhodococcus* spp. (Maeda *et al.*, 1995; Kosono *et al.*, 1997; Taguchi *et al.*, 2004). In addition to these examples, multiplicities in *cad* genes responsible for 2,4-D and 2,4,5-T degradation were observed in the present study.

### Acquisition of the *cad1* cluster via horizontal gene transfer

*cad1* and *cad2* clusters appear to be of distinct origins, with the former being acquired via horizontal gene transfer based on analyses of GC contents, codon usage, and phylogenetic properties (Fig. 1, 2, and S1 and Table S4). *tfdBaFRDEC* present upstream of the *cad1* cluster is presumed to have been acquired via horizontal gene transfer with the *cad1* cluster. On the other hand, the *cad2* cluster and *tfdA*α were evolutionarily acquired by *Bradyrhizobium* without any recent horizontal transfer. Therefore, *Bradyrhizobium* sp. RD5-C2 evolved from a non-2,4-D degrader that harbored the *cad2* cluster via the acquisition of the *cad1* cluster, thereby becoming a 2,4-D degrader. The GC contents of the *car*-I (52.6–62.7%) and *car*-II (59.4–71.3%) gene clusters in *Norosphingomonas* sp. KA1 were previously reported to differ (Urata *et al.*, 2006) from those of the *cad1* and *cad2* clusters. The GC contents of the *nid* (AF546904, 63.0–67.6%) and *pdo* (AF546905, 62.5–66.9%) clusters in *Mycobacterium* sp. S65 were similar. The duplication of degradation genes within a bacterial genome may lead to similarities in the GC contents of these genes. Differences in the GC contents of degradation genes may be attributed to the process of gene acquisition.

*Bradyrhizobium* sp. RD5-C2 was isolated from soil with no previous history of exposure to 2,4-D or 2,4,5-T. *Bradyrhizobium* sp. HW13 and *Bradyrhizobium* sp. BTH, which contain the homologous gene of *cadA1*, were isolated from pristine soil with no 2,4-D contamination in Hawaii and Canada, respectively (Kamagata *et al.*, 1997). This suggests that chlorophenoxyacetic acids are not a selective pressure for the acquisition of the *cad1* cluster in 2,4-D degraders. Additionally, no *Bradyrhizobium* strain that harbors the homologous *cadA1* gene from 2,4-D-contaminated environments has been reported to date. Although there is no experimental data to exclude the possibility that the acquisition of the *cad1* cluster occurred under the selective pressure of 2,4-D in enrichment processes, the acquisition of the *cad1* cluster via horizontal gene transfer may be related to unknown factors, except for chlorophenoxyacetic acids, in the original soil in which the microbes evolved.

### Original substrates of *cad* clusters

The isolation source of *Bradyrhizobium* sp. RD5-C2 indicated that 2,4-D and 2,4,5-T were not the original substrates of the *cad1* and *cad2* clusters, although *cadA1* was strongly induced by 2,4-D and both clusters were capable of degrading 2,4-D and 2,4,5-T. The presence of the homologs of the *cad2* cluster in *Bradyrhizobium* strains (Fig. 2) indicates that these enzymes play important roles in the oxygenation of other unknown natural compound(s). The multiplication of degradation genes generally enables an organism to utilize novel carbon and energy sources for survival. *Bradyrhizobium* sp. RD5-C2 may exhibit additional degrading activity for a wider range of compound(s) by acquiring the *cad1* cluster.

### *Cad* proteins in two clusters

Although CadA1 belongs to a distinct clade with CadA in *Bradyrhizobium* sp. HW13, it shares a common ancestor with the CadA2 lineage (Fig. 2A). Similarly, the two CadB of *Bradyrhizobium* sp. RD5-C2 fell into a branch that contained no known oxygenases of other substances. These phylogenies indicate that the *cadA* and *cadB* genes provide chlorophenoxyacetic acid substrate specificity. In contrast, the CadC1 protein was located in different branches of CadC2 in the phylogenetic tree (Fig. 2C). Since *cadC* and its homologous genes are predicted to encode ferredoxin components, they do not possess high substrate specificity.

Regarding regulators, the two *cad* clusters were located in different contigs and both of them contained *cadR*. CadR1 and CadR2 exhibited distinct amino acid sequences, indicating that the expression of the two *cadABCK* genes was independently regulated. Based on the inhibition of 2,4-D degradation by the *cadR1* deletion (Fig. 4), we conclude that *cadR1* is necessary for the downstream expression of genes, and CadR1 appears to induce downstream *cad1*-degrading genes in the presence of 2,4-D. *cadR1* is presumed to be specifically adapted to induce the expression of downstream degradation genes in response to 2,4-D, and this is supported by the phylogenetic property of CadR1, which belongs to a distinct clade with CadR in 2,4-D degraders (Fig. 2E). The GC content of *cadK1* is distinct from that of the remaining *cad1* genes, but is similar to that of *cad2* genes, implying that *cadK1* may have been inserted into the region between *cadB1* and *cadC1* after the acquisition of *cadR1A1B1C1*.

## Conclusion

The present results demonstrated that *Bradyrhizobium* sp. RD5-C2 possessed two distinct *cad* clusters, which have the ability to degrade chlorophenoxyacetic acids when expressed. The degradation of these compounds by *Bradyrhizobium* sp. RD5-C2 is primarily mediated by the *cad1* cluster, which is induced at high levels. The results of the phylogenetic analysis imply that *Bradyrhizobium* sp. RD5-C2 evolved from a non-2,4-D degrader that harbored the *cad2* cluster and subsequently acquired the *cad1* cluster via horizontal gene transfer, thereby becoming a 2,4-D degrader.

## Citation

Hayashi, S., Tanaka, S., Takao, S., Kobayashi, S., Suyama, K., and Itoh, K. (2021) Multiple Gene Clusters and Their Role in the Degradation of Chlorophenoxyacetic Acids in *Bradyrhizobium* sp. RD5-C2 Isolated from Non-Contaminated Soil. *Microbes Environ ***36**: ME21016.

https://doi.org/10.1264/jsme2.ME21016

## Supplementary Material

Supplementary Material 1

Supplementary Material 2

## Figures and Tables

**Table 1.** *Bradyrhizobium* strains used in the present study.

Strain	Characteristics	Source or reference
*Bradyrhizobium* sp.		
RD5-C2	Wild-type strain, Tc^R^, Km^S^	(Itoh *et al.*, 2000)
RD5-C2ΔcadA1	In-frame disruption mutant of *cadA1* of RD5-C2	This study
RD5-C2ΔcadA2	In-frame disruption mutant of *cadA2* of RD5-C2	This study
RD5-C2ΔtfdAα	In-frame disruption mutant of *tfdAα* of RD5-C2	This study
RD5-C2ΔcadA1ΔcadA2	In-frame disruption mutant of *cadA1* and *cadA2* of RD5-C2	This study
RD5-C2ΔcadA1ΔcadA2tfdAα	In-frame disruption mutant of *cadA1*, *cadA2*, and *tfdAα* of RD5-C2	This study
RD5-C2ΔcadR1	In-frame disruption mutant of *cadR1* of RD5-C2	This study
RD5-C2ΔcadA1/BBR2-*cadA1*	RD5-C2ΔcadA1 harboring pBBR2-C2cadA1pro-cadA1, Km^r^	This study
RD5-C2ΔcadA1/BBR2	RD5-C2ΔcadA1 harboring pBBR1MCS2_START (empty vector), Km^r^	This study
*Bradyrhizobium* *elkanii*		
USDA94	Wild-type strain, Tc^R^, Km^S^	(Minamisawa *et al.*, 2002)
USDA94BBR2C2cad1ABKC	USDA94 harboring pBBR2-C2cad1ABCK, Km^r^	This study
USDA94BBR2C2cad2ABCK	USDA94 harboring pBBR2-C2cad2ABCK, Km^r^	This study
USDA94BBR2	USDA94 harboring pBBR1MCS2_START (empty vector), Km^r^	This study

Km: Kanamycin, Tc: Tetracycline

## References

[B1] Collier, L.S., Gaines, G.L., 3rd, and Neidle, E.L. (1998) Regulation of benzoate degradation in *Acinetobacter* sp. strain ADP1 by BenM, a LysR-type transcriptional activator. J Bacteriol 180: 2493–2501. 9573203 10.1128/jb.180.9.2493-2501.1998 PMC107193

[B2] Danganan, C.E., Ye, R.W., Daubaras, D.L., Xun, L., and Chakrabarty, A.M. (1994) Nucleotide sequence and functional analysis of the genes encoding 2,4,5-trichlorophenoxyacetic acid oxygenase in *Pseudomonas cepacia* AC1100. Appl Environ Microbiol 60: 4100–4106. 7527626 10.1128/aem.60.11.4100-4106.1994 PMC201942

[B3] Dennis, J.J., and Zylstra, G.J. (2004) Complete sequence and genetic organization of pDTG1, the 83 kilobase naphthalene degradation plasmid from *Pseudomonas putida* strain NCIB 9816-4. J Mol Biol 341: 753–768. 15288784 10.1016/j.jmb.2004.06.034

[B4] Don, R.H., and Pemberton, J.M. (1981) Properties of six pesticide degradation plasmids isolated from *Alcaligenes paradoxus* and *Alcaligenes eutrophus*. J Bacteriol 145: 681–686. 6257648 10.1128/jb.145.2.681-686.1981 PMC217166

[B5] Donald, D.B., Cessna, A.J., Sverko, E., and Glozier, N.E. (2007) Pesticides in surface drinking-water supplies of the northern Great Plains. Environ Health Perspect 115: 1183–1191. 17687445 10.1289/ehp.9435 PMC1940079

[B6] Gevers, D., Vandepoele, K., Simillon, C., and Van de Peer, Y. (2004) Gene duplication and biased functional retention of paralogs in bacterial genomes. Trends Microbiol 12: 148–154. 15116722 10.1016/j.tim.2004.02.007

[B7] Golovleva, L.A., Pertsova, R.N., Evtushenko, L.I., and Baskunov, B.P. (1990) Degradation of 2,4,5-trichlorophenoxyacetic acid by a *Nocardioides simplex* culture. Biodegradation 1: 263–271. 1368472 10.1007/BF00119763

[B8] Gomada, M., Inouye, S., Imaishi, H., Nakazawa, A., and Nakazawa, T. (1992) Analysis of an upstream regulatory sequence required for activation of the regulatory gene xylS in xylene metabolism directed by the TOL plasmid of *Pseudomonas putida*. Mol Gen Genet 233: 419–426. 1620097 10.1007/BF00265439

[B9] Hayashi, S., Sano, T., Suyama, K., and Itoh, K. (2016) 2,4-Dichlorophenoxyacetic acid (2,4-D)- and 2,4,5-trichlorophenoxyacetic acid (2,4,5-T)-degrading gene cluster in the soybean root-nodulating bacterium *Bradyrhizobium elkanii* USDA94. Microbiol Res 188: 62–71. 27296963 10.1016/j.micres.2016.04.014

[B10] Huong, N.L., Itoh, K., Miyamoto, M., Suyama, K., and Yamamoto, H. (2007a) Chlorophenol hydroxylase activity encoded by TfdB from 2,4-dichlorophenoxyacetic acid (2,4-D)-degrading *Bradyrhizobium* sp. strain RD5-C2. Biosci Biotechnol Biochem 71: 1691–1696. 17617713 10.1271/bbb.70106

[B11] Huong, N.L., Itoh, K., and Suyama, K. (2007b) Diversity of 2,4-dichlorophenoxyacetic acid (2,4-D) and 2,4,5-trichlorophenoxyacetic acid (2,4,5-T)-degrading bacteria in Vietnamese soils. Microbes Environ 22: 243–256. 10.1264/jsme2.23.142 21558700

[B12] Inoue, K., Habe, H., Yamane, H., and Nojiri, H. (2006) Characterization of novel carbazole catabolism genes from gram-positive carbazole degrader *Nocardioides aromaticivorans* IC177. Appl Environ Microbiol 72: 3321–3329. 16672473 10.1128/AEM.72.5.3321-3329.2006 PMC1472339

[B13] Itoh, K., Kameda, R., Momoda, Y., Sumida, Y., Kamagata, Y., Suyama, K., and Yamamoto, H. (2000) Presence of 2,4-D-catabolizing bacteria in a Japanese arable soil that belong to BANA (Bradyrhizobium-Agromonas-Nitrobacter-Afipia) cluster in α-Proteobacteria. Microbes Environ 15: 113–117.

[B14] Itoh, K., Kanda, R., Sumita, Y., Kim, H., Kamagata, Y., Suyama, K., et al. (2002) *tfdA*-like genes in 2,4-dichlorophenoxyacetic acid-degrading bacteria belonging to the Bradyrhizobium-Agromonas-Nitrobacter-Afipia cluster in alpha-Proteobacteria. Appl Environ Microbiol 68: 3449–3454. 12089027 10.1128/AEM.68.7.3449-3454.2002 PMC126798

[B15] Itoh, K., Tashiro, Y., Uobe, K., Kamagata, Y., Suyama, K., and Yamamoto, H. (2004) Root nodule Bradyrhizobium spp. harbor *tfdAalpha* and *cadA*, homologous with genes encoding 2,4-dichlorophenoxyacetic acid-degrading proteins. Appl Environ Microbiol 70: 2110–2118. 15066803 10.1128/AEM.70.4.2110-2118.2004 PMC383140

[B16] Iwasaki, T., Miyauchi, K., Masai, E., and Fukuda, M. (2006) Multiple-subunit genes of the aromatic-ring-hydroxylating dioxygenase play an active role in biphenyl and polychlorinated biphenyl degradation in *Rhodococcus* sp. strain RHA1. Appl Environ Microbiol 72: 5396–5402. 16885291 10.1128/AEM.00298-06 PMC1538765

[B17] Kamagata, Y., Fulthorpe, R.R., Tamura, K., Takami, H., Forney, L.J., and Tiedje, J.M. (1997) Pristine environments harbor a new group of oligotrophic 2,4-dichlorophenoxyacetic acid-degrading bacteria. Appl Environ Microbiol 63: 2266–2272. 9172346 10.1128/aem.63.6.2266-2272.1997 PMC168519

[B18] Kellogg, S.T., Chatterjee, D.K., and Chakrabarty, A.M. (1981) Plasmid-assisted molecular breeding: new technique for enhanced biodegradation of persistent toxic chemicals. Science 214: 1133–1135. 7302584 10.1126/science.7302584

[B19] Kilbane, J.J., Chatterjee, D.K., Karns, J.S., Kellogg, S.T., and Chakrabarty, A.M. (1982) Biodegradation of 2,4,5-trichlorophenoxyacetic acid by a pure culture of *Pseudomonas cepacia*. Appl Environ Microbiol 44: 72–78. 7125648 10.1128/aem.44.1.72-78.1982 PMC241970

[B20] Kim, S.J., Kweon, O., Freeman, J.P., Jones, R.C., Adjei, M.D., Jhoo, J.W., et al. (2006) Molecular cloning and expression of genes encoding a novel dioxygenase involved in low- and high-molecular-weight polycyclic aromatic hydrocarbon degradation in *Mycobacterium vanbaalenii* PYR-1. Appl Environ Microbiol 72: 1045–1054. 16461648 10.1128/AEM.72.2.1045-1054.2006 PMC1392982

[B21] Kitagawa, W., Miyauchi, K., Masai, E., and Fukuda, M. (2001) Cloning and characterization of benzoate catabolic genes in the gram-positive polychlorinated biphenyl degrader *Rhodococcus* sp. strain RHA1. J Bacteriol 183: 6598–6606. 11673430 10.1128/JB.183.22.6598-6606.2001 PMC95491

[B22] Kitagawa, W., Takami, S., Miyauchi, K., Masai, E., Kamagata, Y., Tiedje, J.M., and Fukuda, M. (2002) Novel 2,4-dichlorophenoxyacetic acid degradation genes from oligotrophic *Bradyrhizobium* sp. strain HW13 isolated from a pristine environment. J Bacteriol 184: 509–518. 11751829 10.1128/JB.184.2.509-518.2002 PMC139574

[B23] Kosono, S., Maeda, M., Fuji, F., Arai, H., and Kudo, T. (1997) Three of the seven *bphC* genes of *Rhodococcus erythropolis* TA421, isolated from a termite ecosystem, are located on an indigenous plasmid associated with biphenyl degradation. Appl Environ Microbiol 63: 3282–3285. 9251216 10.1128/aem.63.8.3282-3285.1997 PMC168627

[B24] Kumar, S., Stecher, G., and Tamura, K. (2016a) MEGA7: Molecular evolutionary genetics analysis version 7.0 for bigger datasets. Mol Biol Evol 33: 1870–1874. 27004904 10.1093/molbev/msw054 PMC8210823

[B25] Kumar, A., Trefault, N., and Olaniran, A.O. (2016b) Microbial degradation of 2,4-dichlorophenoxyacetic acid: Insight into the enzymes and catabolic genes involved, their regulation and biotechnological implications. Crit Rev Microbiol 42: 194–208. 25058513 10.3109/1040841X.2014.917068

[B26] Kurtz, S., Phillippy, A., Delcher, A.L., Smoot, M., Shumway, M., Antonescu, C., and Salzberg, S.L. (2004) Versatile and open software for comparing large genomes. Genome Biol 5: R12. 14759262 10.1186/gb-2004-5-2-r12 PMC395750

[B27] Laemmli, C.M., Leveau, J.H., Zehnder, A.J., and van der Meer, J.R. (2000) Characterization of a second *tfd* gene cluster for chlorophenol and chlorocatechol metabolism on plasmid pJP4 in *Ralstonia eutropha* JMP134(pJP4). J Bacteriol 182: 4165–4172. 10894723 10.1128/jb.182.15.4165-4172.2000 PMC101896

[B28] Leveau, J.H., Konig, F., Fuchslin, H., Werlen, C., and Van Der Meer, J.R. (1999) Dynamics of multigene expression during catabolic adaptation of *Ralstonia eutropha* JMP134 (pJP4) to the herbicide 2,4-dichlorophenoxyacetate. Mol Microbiol 33: 396–406. 10411755 10.1046/j.1365-2958.1999.01483.x

[B29] Liu, T., and Chapman, P.J. (1984) Purification and properties of a plasmid-encoded 2,4-dichlorophenol hydroxylase. FEBS Lett 173: 314–318. 6745439 10.1016/0014-5793(84)80797-8

[B30] Maeda, M., Chung, S.Y., Song, E., and Kudo, T. (1995) Multiple genes encoding 2,3-dihydroxybiphenyl 1,2-dioxygenase in the gram-positive polychlorinated biphenyl-degrading bacterium *Rhodococcus erythropolis* TA421, isolated from a termite ecosystem. Appl Environ Microbiol 61: 549–555. 7574595 10.1128/aem.61.2.549-555.1995 PMC167317

[B31] Mazodier, P., Petter, R., and Thompson, C. (1989) Intergeneric conjugation between *Escherichia coli* and *Streptomyces species*. J Bacteriol 171: 3583–3585. 2656662 10.1128/jb.171.6.3583-3585.1989 PMC210093

[B32] Minamisawa, K., Isawa, T., Nakatsuka, Y., and Ichikawa, N. (1998) New *Bradyrhizobium japonicum* strains that possess high copy numbers of the repeated sequence RS alpha. Appl Environ Microbiol 64: 1845–1851. 9572961 10.1128/aem.64.5.1845-1851.1998 PMC106240

[B33] Minamisawa, K., Itakura, M., Suzuki, M., Ichige, K., Isawa, T., Yuhashi, K., and Mitsui, H. (2002) Horizontal transfer of nodulation genes in soils and microcosms from *Bradyrhizobium japonicum* to *B. elkanii*. Microbes Environ 17: 82–90.

[B34] Müller, T.A., Byrde, S.M., Werlen, C., van der Meer, J.R., and Kohler, H.P. (2004) Genetic analysis of phenoxyalkanoic acid degradation in *Sphingomonas herbicidovorans* MH. Appl Environ Microbiol 70: 6066–6075. 15466552 10.1128/AEM.70.10.6066-6075.2004 PMC522092

[B35] Neidle, E.L., Hartnett, C., Ornston, L.N., Bairoch, A., Rekik, M., and Harayama, S. (1991) Nucleotide sequences of the Acinetobacter calcoaceticus *benABC* genes for benzoate 1,2-dioxygenase reveal evolutionary relationships among multicomponent oxygenases. J Bacteriol 173: 5385–5395. 1885518 10.1128/jb.173.17.5385-5395.1991 PMC208249

[B36] Nielsen, T.K., Xu, Z., Gozdereliler, E., Aamand, J., Hansen, L.H., and Sorensen, S.R. (2013) Novel insight into the genetic context of the *cadAB* genes from a 4-chloro-2-methylphenoxyacetic acid-degrading Sphingomonas. PLoS One 8: e83346. 24391756 10.1371/journal.pone.0083346 PMC3877037

[B37] Nojiri, H., Tsuda, M., Fukuda, M., and Kamagata, Y. (2014) *Biodegradative Bacteria: How Bacteria Degrade, Survive, Adapt, and Evolve*. Tokyo, Japan: Springer.

[B38] Obranić, S., Babić, F., and Maravić-Vlahoviček, G. (2013) Improvement of pBBR1MCS plasmids, a very useful series of broad-host-range cloning vectors. Plasmid 70: 263–267. 23583732 10.1016/j.plasmid.2013.04.001

[B39] Parks, D.H., Imelfort, M., Skennerton, C.T., Hugenholtz, P., and Tyson, G.W. (2015) CheckM: assessing the quality of microbial genomes recovered from isolates, single cells, and metagenomes. Genome Res 25: 1043–1055. 25977477 10.1101/gr.186072.114 PMC4484387

[B40] Perkins, E.J., Gordon, M.P., Caceres, O., and Lurquin, P.F. (1990) Organization and sequence analysis of the 2,4-dichlorophenol hydroxylase and dichlorocatechol oxidative operons of plasmid pJP4. J Bacteriol 172: 2351–2359. 2185214 10.1128/jb.172.5.2351-2359.1990 PMC208869

[B41] Rice, J.F., Menn, F.M., Hay, A.G., Sanseverino, J., and Sayler, G.S. (2005) Natural selection for 2,4,5-trichlorophenoxyacetic acid mineralizing bacteria in agent orange contaminated soil. Biodegradation 16: 501–512. 15865343 10.1007/s10532-004-6186-8

[B42] Sambrook, J., and Russell, D. (2001) *Molecular Cloning: A Laboratory Manual*. Cold Spring Harbor, NY: Cold Spring Harbor Laboratory Press.

[B43] Schäfer, A., Tauch, A., Jäger, W., Kalinowski, J., Thierbach, G., and Pühler, A. (1994) Small mobilizable multi-purpose cloning vectors derived from the *Escherichia coli* plasmids pK18 and pK19: selection of defined deletions in the chromosome of *Corynebacterium glutamicum*. Gene 145: 69–73. 8045426 10.1016/0378-1119(94)90324-7

[B44] Serbent, M.P., Rebelo, A.M., Pinheiro, A., Giongo, A., and Tavares, L.B.B. (2019) Biological agents for 2,4-dichlorophenoxyacetic acid herbicide degradation. Appl Microbiol Biotechnol 103: 5065–5078. 31044311 10.1007/s00253-019-09838-4

[B45] Shimojo, M., Kawakami, M., and Amada, K. (2009) Analysis of genes encoding the 2,4-dichlorophenoxyacetic acid-degrading enzyme from *Sphingomonas agrestis* 58-1. J Biosci Bioeng 108: 56–59. 19577193 10.1016/j.jbiosc.2009.02.018

[B46] Sho, M., Hamel, C., and Greer, C.W. (2004) Two distinct gene clusters encode pyrene degradation in *Mycobacterium* sp. strain S65. FEMS Microbiol Ecol 48: 209–220. 19712404 10.1016/j.femsec.2004.01.011

[B47] Stover, C.K., Pham, X.Q., Erwin, A.L., Mizoguchi, S.D., Warrener, P., Hickey, M.J., et al. (2000) Complete genome sequence of *Pseudomonas aeruginosa* PAO1, an opportunistic pathogen. Nature 406: 959–964. 10984043 10.1038/35023079

[B48] Taguchi, K., Motoyama, M., and Kudo, T. (2004) Multiplicity of 2,3-dihydroxybiphenyl dioxygenase genes in the gram-positive polychlorinated biphenyl degrading bacterium *Rhodococcus rhodochrous* K37. Biosci Biotechnol Biochem 68: 787–795. 15118304 10.1271/bbb.68.787

[B49] Urata, M., Uchimura, H., Noguchi, H., Sakaguchi, T., Takemura, T., Eto, K., et al. (2006) Plasmid pCAR3 contains multiple gene sets involved in the conversion of carbazole to anthranilate. Appl Environ Microbiol 72: 3198–3205. 16672458 10.1128/AEM.72.5.3198-3205.2006 PMC1472349

[B50] Xun, L., and Wagnon, K.B. (1995) Purification and properties of component B of 2,4,5-trichlorophenoxyacetate oxygenase from *Pseudomonas cepacia* AC1100. Appl Environ Microbiol 61: 3499–3502. 16535134 10.1128/aem.61.9.3499-3502.1995 PMC1388588

[B51] Zhang, C., and Anderson, A.J. (2012) Multiplicity of genes for aromatic ring-hydroxylating dioxygenases in *Mycobacterium isolate* KMS and their regulation. Biodegradation 23: 585–596. 22307885 10.1007/s10532-012-9535-z

[B52] Zhou, N.Y., Fuenmayor, S.L., and Williams, P.A. (2001) *nag* genes of *Ralstonia* (formerly *Pseudomonas*) sp. strain U2 encoding enzymes for gentisate catabolism. J Bacteriol 183: 700–708. 11133965 10.1128/JB.183.2.700-708.2001 PMC94927

[B53] Zylstra, G.J., and Gibson, D.T. (1989) Toluene degradation by *Pseudomonas putida* F1. nucleotide sequence of the *todC1C2BADE* genes and their expression in *Escherichia coli*. J Biol Chem 264: 14940–14946. 2670929

