# Supplementary material for: Multiple Gene Clusters and Their Role in the Degradation of Chlorophenoxyacetic Acids in Bradyrhizobium sp. RD5-C2 Isolated from Non-Contaminated Soil
Source: Microbes Environ. 2021 Sep 11;36(3):ME21016. doi: 10.1264/jsme2.ME21016 (PMC8446748; doi:10.1264/jsme2.ME21016)
Supplement: Supplementary file 1 — Supplementary Material 1 [file 36_21016_s1.pdf]

Supplemental Information

Multiple gene clusters and their role in the degradation of chlorophenoxyacetic acids in *Bradyrhizobium* sp. RD5-C2 isolated from non-contaminated soil

Shohei Hayashi, Sho Tanaka, Soichiro Takao, Shinnosuke Kobayashi, Kousuke Suyama, Kazuhito Itoh

Supplemental Tables

Table S1. Genes used in this study. (on the Excel file)

Table S2. Plasmids used in this study.

Table S3. Primers used in this study.

Table S4. Codon usages (%) of housekeeping and 2,4-D-degrading genes. (on the Excel file)

Supplemental Figures

Fig. S1 Hierarchical cluster of the codon usages of housekeeping and 2,4-D-degrading genes.

Fig. S2. Genetic organization to the *tfd* genes and *cad1* cluster.

Fig. S3. Degradation of 2,4-dichlorophenoxyacetic acid (2,4-D) (A) and 2,4,5-trichlorophenoxyacetic acid (2,4,5-T) (B) by *tfdAα* deletion mutants of *Bradyrhizobium* sp. RD5-C2.

Fig. S4. Degradation of 2,4-dichlorophenoxyacetic acid (2,4-D) by *cadA1* complementary strain of the

deletion mutant of *Bradyrhizobium* sp. RD5-C2.

Supplemental references

Supplemental Tables

Table S2. Plasmids used in this study.

Plasmid	Characteristic	Source or reference
pBBR1MCS2_START	expression vector in *Bradyrhizobium*, Km^r, *lacZα*, mob^+	(Obranic *et al.*, 2013)
pBBR2-C2cad1ABKC	pBBR1MCS2_START containing *cadA1B1K1C1* from *Bradyrhizobium* sp. RD5-C2	This study
pBBR2-C2cad2ABCK	pBBR1MCS2_START containing *cadA2B2C2K2* from *Bradyrhizobium* sp. RD5-C2	This study
pBBR2-C2cadA1pro-cadA1	pBBR1MCS2_START containing *cadA1* and *cadA1* promoter region from *Bradyrhizobium* sp. RD5-C2	This study
pK18mob	suicide vector in *Bradyrhizobium*, Km^r, mob^+	(Schäfer *et al.*, 1994)
pK18mob-C2cadA1updw	pK18mob containing both approximately 2-kb upstream and downstream regions of *cadA1*	This study
pK18mob-C2cadA2updw	pK18mob containing both approximately 2-kb upstream and downstream regions of *cadA2*	This study
pK18mob-C2tfdAαupdw	pK18mob containing both approximately 2-kb upstream and downstream regions of *tfdAα*	This study

pK18mobsacB	suicide vector in *Bradyrhizobium*, Km^r, mob^+, *sacB*	(Schäfer *et al.*, 1994)
pK18mobsacB-C2cadA2updw	pK18mobsacB containing both approximately 2-kb upstream and downstream regions of *cadA2*	This study
pK18mobsacB-C2tfdAαupdw	pK18mobsacB containing both approximately 2-kb upstream and downstream regions of *tfdAα*	This study
pK18mobsacB-C2cadR1updw	pK18mobsacB containing both approximately 2-kb upstream and downstream regions of *cadR1*	This study

Km: Kanamycin

Table S3. Primers used in this study.

Primer	Sequence (5′-3′)	Position	Reference
C2cad1A-F-Nde	AGATCCATATGGACGTTGAACGCCTGAACACGC	1–25 in *cadA1*	This study
C2cad1C-R-Bam	ATCGGGATCCGATACCTATTCCGTATGCCCCCCT	25–2 downstream from *cadC1*	This study
BBR2+C2cad2-F	TCACACAGGAAACACATATGAACGCTGCTTCGCAG	1–18 in *cadA2*	This study
BBR2+C2cad2-R	CTTGGCGTAATCATGGTTGGTACGAGCAGTCCGGC	22–4 downstream from *cadK2*	This study
C2cad2+BBR2-F	CCGGACTGCTCGTACCAACCATGATTACGCCAAGC	4–21 in *lacZα* on pBBR1MCS2_START	This study
C2cad2+BBR2-R	TGCGAAGCAGCGTTCATATGTGTTTCCTGTGTGAA	4–-18 in *lacZα* on pBBR1MCS2_START	This study
Dcad1Aup5-Eco	GCAGAATTCCTTCGAAGGCGGCAAGTATG	1879–1860 upstream from *cadA1*	This study
Dcad1Aup3-Xba	GACTCTAGACCACGTTCCCTCAAAGATCG	132–113 in *cadA1*	This study
Dcad1Adw5-Xba	GCCTCTAGAGGCCCAAGGCAGGGATAC	1144–1161 in *cadA1*	This study
Dcad1Adw3-Hind	AGCAAGCTTCACTGGCCCGATTGCTATTC	1817–1798 downstream from *cadA1*	This study

DcadAup5-Kpn	GATGGTACCGCCAATACTCGTTCGACATC	1826–1807 upstream from *cadA2*	(Hayashi *et al.*, 2016)
DcadAup3-Xba	CCATCTAGATGAAATTCCACGTGGCCTCG	166–147 in *cadA2*	(Hayashi *et al.*, 2016)
DcadAdw5-Xba	GTTTCTAGAGCGGCGTCGCGGAGGTCGGC	1205–1224 in *cadA2*	(Hayashi *et al.*, 2016)
DcadAdw3-Hind	GGCAAGCTTCGCTGCCGCGATCACGAAGG	1881–1862 downstream from *cadA2*	(Hayashi *et al.*, 2016)
DtfdAαup5-Kpn	GACGGTACCGGTCTGGCCGACGATGTAGG	1775–1756 upstream from *tfdAα*	(Hayashi *et al.*, 2016)
DtfdAαup3-Xba	GGATCTAGAGCACGGCGTATTTGTCCATG	127–108 in *tfdAα*	(Hayashi *et al.*, 2016)
DtfdAαdw5-Xba	GCATCTAGACGTGATGTGGGACAACCGCC	762–781 in *tfdAα*	(Hayashi *et al.*, 2016)
DtfdAαdw3-Hind	TTGAAGCTTCCGACCAGCGTCAGCTTCAG	1798–1779 downstream from *tfdAα*	(Hayashi *et al.*, 2016)
DcadAup5-Bam	GATGGATCCGCCAATACTCGTTCGACATC	1826–1807 upstream from *cadA2*	(Hayashi *et al.*, 2016)
DtfdAaup5-Bam	GACGGATCCGGTCTGGCCGACGATGTAGG	1775–1756 upstream from *tfdAα*	This study
DC2cad1up5-Hin	TTTCGAAGCTTGGAGCGCAGATGTCGAACTTG	1949–1929 upstream from *cadR1*	This study
DC2cad1up3-Xba	TGAGTTCTAGAGCGAGATGGTCGGTCCATGAACT	101–79 in *cadR1*	This study

DC2cad1Rdw5-Xba	CGCCGTCTAGACGTTAAATTCGGGGTCACG	918–936 in *cadR1*	This study
DC2cad1Rdw3-Bam	CGAGCGGATCCATCCCACCAATGACATGGA	1927–1909 downstream from *cadR1*	This study
C2cad1P-F-Mph	CGCCGATGCATCGTTAAATTCGGGGTCACG	358–340 upstream from *cadA1*	This study
C2cad1A-Bam-R	AACGGATCCGTCCGGTACGTGCAATGTGG	23–3 downstream from *cadA1*	This study
C2cad1A227-f	ACGGACAGGTGGGTTGTTTC	227–246 in *cadA1*	This study
C2cad1A436-r	GCGGCACTTTCACAAGGTC	436–418 in *cadA1*	This study
C2cad2A548-f	CCTTCCTCGACCTCCTCATC	548–567 in *cadA2*	This study
C2cad2A779-r	TGTCCTTCCTGTTCGCCTTC	779–760 in *cadA2*	This study
C2tfdAa308-f	GCACCCACCTGTTCAATCTC	308–327 in *tfdAα*	This study
C2tfdAa488-r	TCGATCTCGGTCTTGGTCTC	488–469 in *tfdAα*	This study

The restriction enzyme sites are underlined.

MCS: multiple cloning site

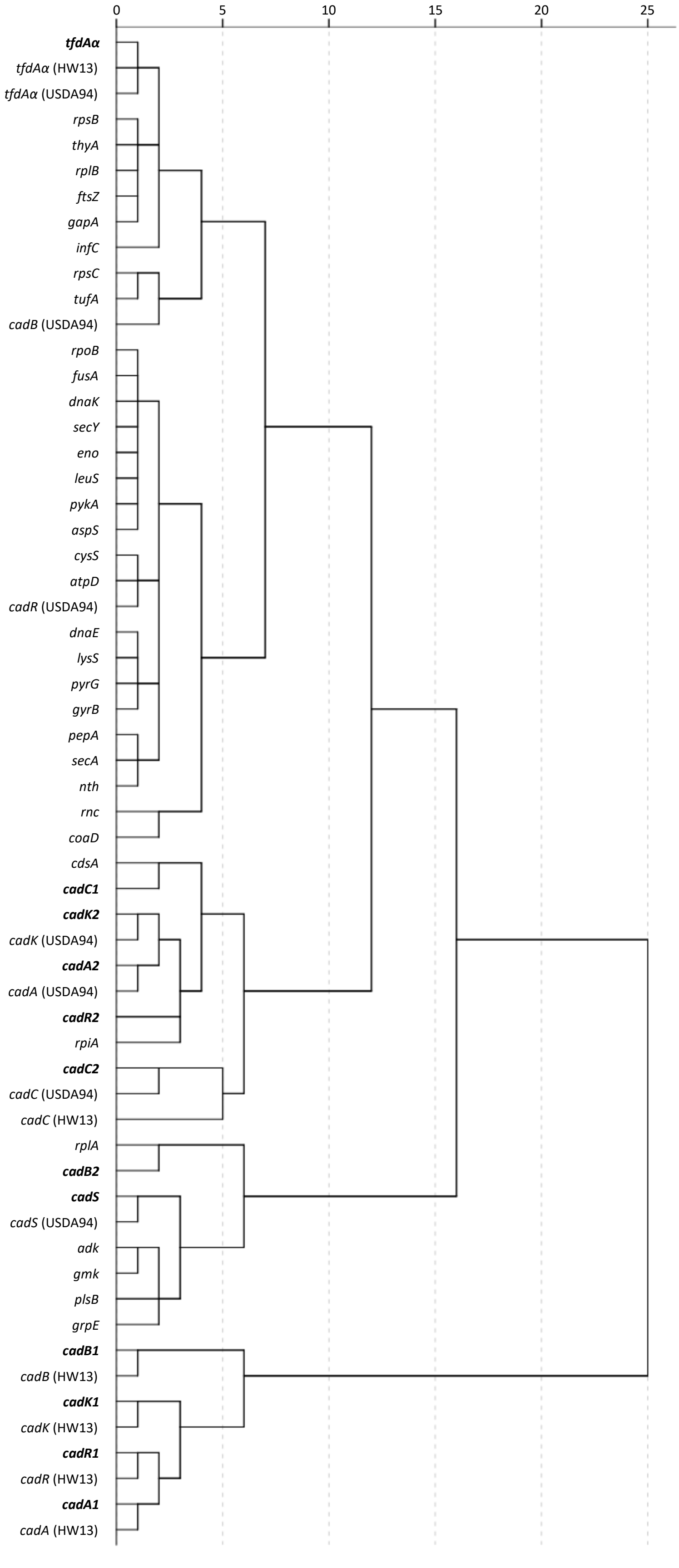

Fig. S1 Hierarchical cluster of the codon usages of housekeeping and 2,4-D-degrading genes.

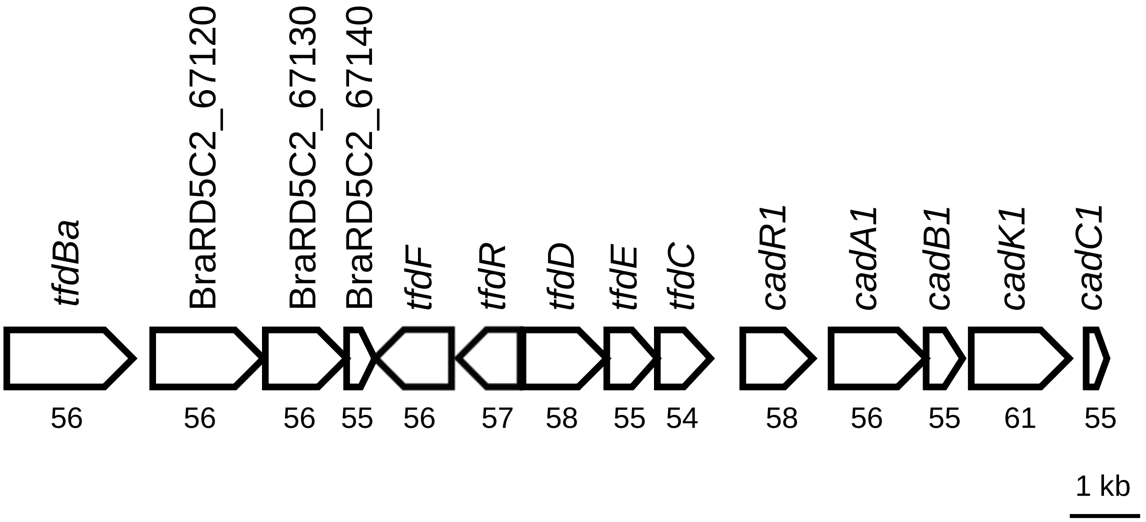

Fig. S2. Genetic organization to the *tfd* genes and *cad1* cluster. Each gene is represented by a large horizontal arrow. The numbers below the genes indicate their GC content (mol %).

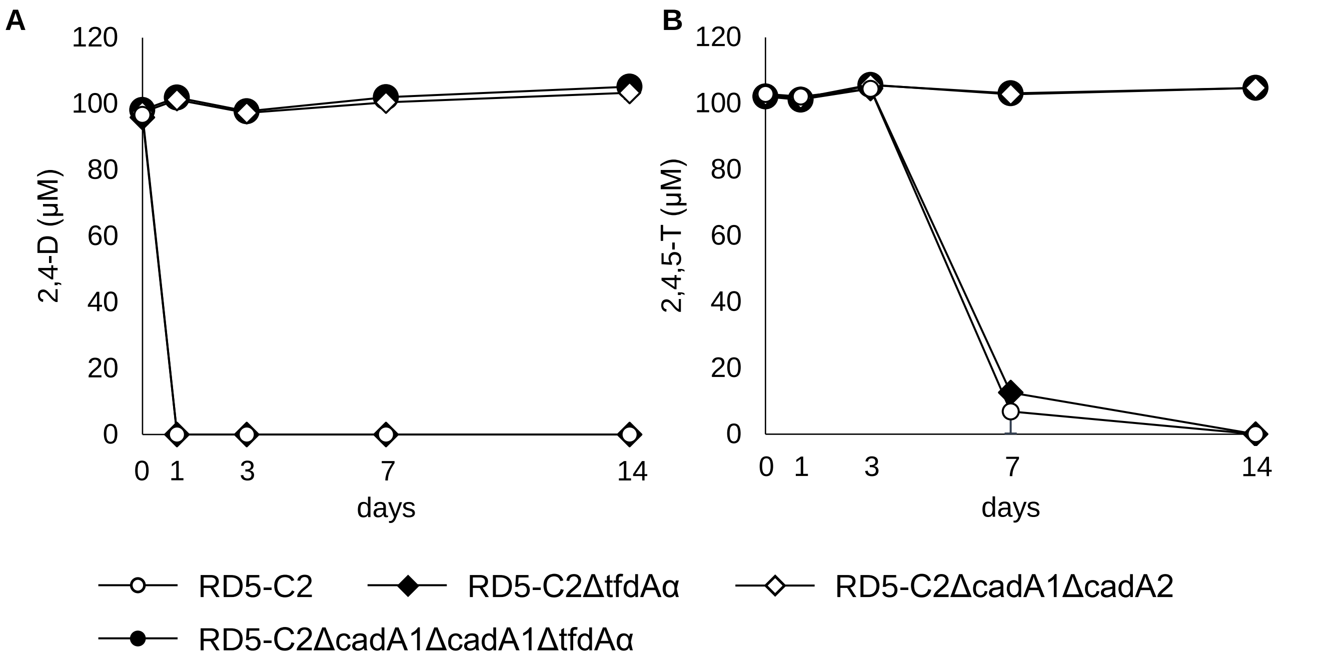

Fig. S3. Degradation of 2,4-dichlorophenoxyacetic acid (2,4-D) (A) and 2,4,5-trichlorophenoxyacetic acid (2,4,5-T) (B) by *tfdAα* deletion mutants of *Bradyrhizobium* sp. RD5-C2. Error bars indicate standard deviations based on triplicate cultures. If not visible, error bars are smaller than symbols.

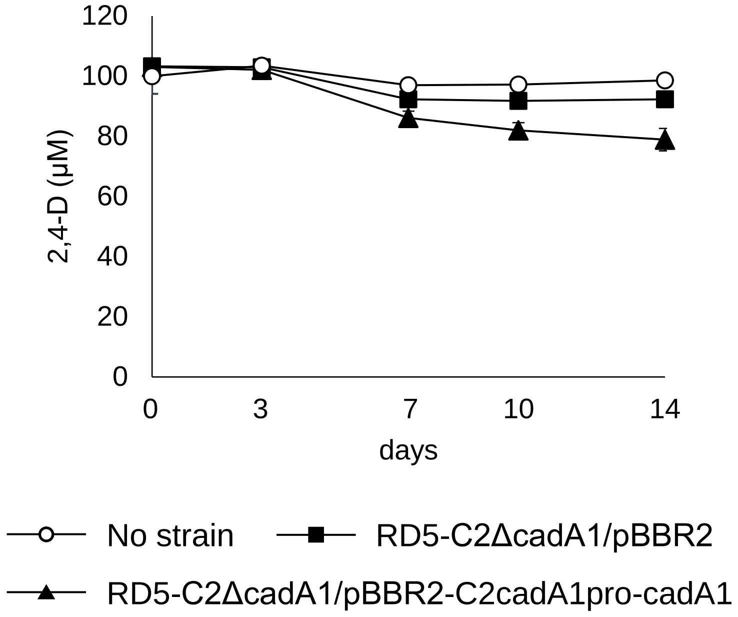

Fig. S4. Degradation of 2,4-dichlorophenoxyacetic acid (2,4-D) by *cadA1* complementary strain of the deletion mutant of *Bradyrhizobium* sp. RD5-C2. Error bars indicate standard deviations based on triplicate cultures. If not visible, error bars are smaller than symbols. The average concentration of 2,4-D degraded by RD5-C2ΔcadA1/pBBR2 after 3 days of incubation was calculated based on the results from two cultures.

Supplemental References

Hayashi, S., Sano, T., Suyama, K. and Itoh, K. (2016) 2,4-Dichlorophenoxyacetic acid (2,4-D)- and 2,4,5-trichlorophenoxyacetic acid (2,4,5-T)-degrading gene cluster in the soybean root-nodulating bacterium *Bradyrhizobium elkanii* USDA94. *Microbiol Res* **188**: 62-71.

Obranic, S., Babic, F. and Maravic-Vlahovicek, G. (2013) Improvement of pBBR1MCS plasmids, a very useful series of broad-host-range cloning vectors. *Plasmid* **70**: 263-267.

Schäfer, A., Tauch, A., Jäger, W., Kalinowski, J., Thierbach, G. and Pühler, A. (1994) Small mobilizable multi-purpose cloning vectors derived from the *Escherichia coli* plasmids pK18 and pK19: selection of defined deletions in the chromosome of *Corynebacterium glutamicum*. *Gene* **145**: 69-73.
